# PI3K/AKT pathway as a key link modulates the multidrug resistance of cancers

**DOI:** 10.1038/s41419-020-02998-6

**Published:** 2020-09-24

**Authors:** Rui Liu, Youwen Chen, Guangzhi Liu, Chenxi Li, Yurong Song, Zhiwen Cao, Wen Li, Jinghong Hu, Cheng Lu, Yuanyan Liu

**Affiliations:** 1 School of Chinese Materia Medica, Beijing University of Chinese Medicine, 100029 Beijing, China; 2 Institute of Basic Research in Clinical Medicine, China Academy of Chinese Medical Sciences, 100700 Beijing, China

**Keywords:** Cancer metabolism, Targeted therapies

## Abstract

Multidrug resistance (MDR) is the dominant challenge in the failure of chemotherapy in cancers. Phosphatidylinositol 3-kinase (PI3K) is a lipid kinase that spreads intracellular signal cascades and regulates a variety of cellular processes. PI3Ks are considered significant causes of chemoresistance in cancer therapy. Protein kinase B (AKT) is also a significant downstream effecter of PI3K signaling, and it modulates several pathways, including inhibition of apoptosis, stimulation of cell growth, and modulation of cellular metabolism. This review highlights the aberrant activation of PI3K/AKT as a key link that modulates MDR. We summarize the regulation of numerous major targets correlated with the PI3K/AKT pathway, which is further related to MDR, including the expression of apoptosis-related protein, ABC transport and glycogen synthase kinase-3 beta (GSK-3β), synergism with nuclear factor kappa beta (NF-κB) and mammalian target of rapamycin (mTOR), and the regulation of glycolysis.

## Facts

- Several possible mechanisms are related to the occurrence and progression of MDR in cancer, including the overexpression of efflux pumps, abnormal tumor microenvironment and other cellular/physiological pathways.
- Activated PI3K/AKT catalyzes the phosphorylation of a series of proteins, promotes tumor cell growth and proliferation, inhibits apoptosis, promotes invasion and metastasis, regulates endothelial cell growth and angiogenesis, and increases the sensitivity to radiation.
- Many types of tumors often carry at least 1 change in PI3K.

## Open questions

- What is the role of the PI3K/AKT pathway in MDR?
- The linkage between PI3KAKT pathway and ABC transporter as well as their synergistic mechanisms need to be studied in-depth to reveal the mechanism of MDR.
- The exact modulation mechanisms of the PI3K/AKT pathway in reversing MDR in a specific manner in tumor cells to lock their growth and metastasis, and induce apoptosis must be deeply investigated in the future.

## Introduction

Although important advances in the field of chemotherapy have lowered the mortality rate for cancer patients, the 5-year survival rate remains gloomy, largely due to intrinsic or acquired mechanisms of resistance to antineoplastic drugs^1^. Multidrug resistance (MDR) describes a phenomenon that leads to resistance to the administered drugs and other drugs with completely different structure and mechanism of actions^2^. There are many mechanisms involved in the generation of MDR. One mechanism is the destruction of apoptosis via the abnormal expression of apoptosis-related factors, which makes cells resistant to drug-induced cell death. A change in the cell cycle process may cause the proliferation of cancer cells and promote resistance^3^. The mechanism classical is to target anticancer drug transport across the cell membrane by increasing the activity of efflux pumps, such as adenosine triphosphate (ATP)-binding cassettes (ABC) transporters^4^. Cancer cells exhibit a special metabolic phenotype—aerobic glycolysis that rapidly transports and consumes glucose to produce ATP and promote drug efflux^5,6^.

**Fig. 1:**
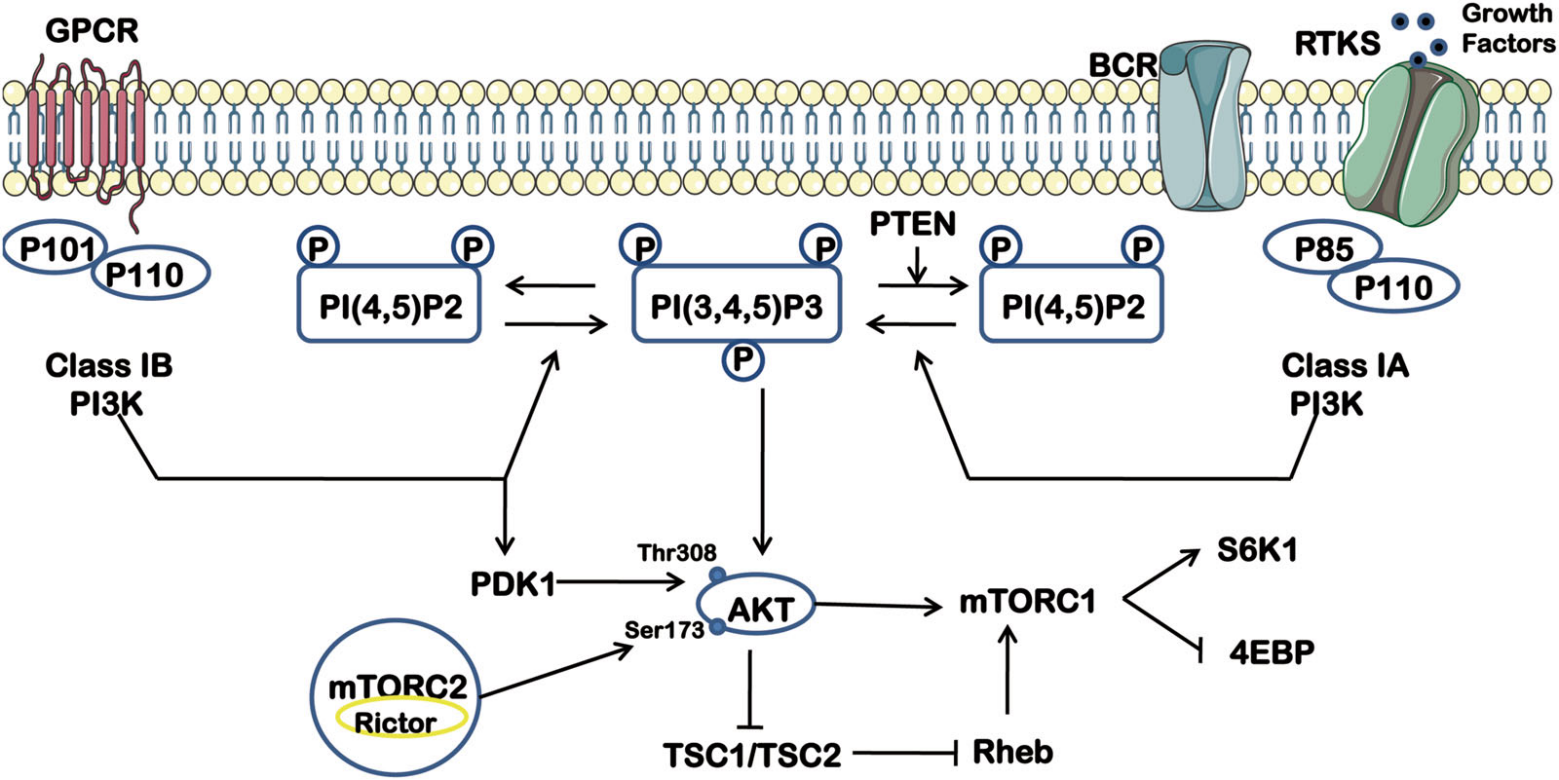
As a major downstream effector of receptor tyrosine kinase (RTK) and G protein-coupled receptors, PI3K activates various downstream effectors by generating phospholipids, transducing signals of various growth factors and cytokines into intracellular information. The main lipid substrate of PTEN is PIP 3 and indeed PTEN acts as a negative regulator of PI3K/AKT signaling. Among the upstream signaling networks, Akt inactivate TSC1/2 and activate mTORC1. mTORC2 directly phosphorylates Akt at S473 residue leading to its complete activation. This activation of the PI3K/Akt pathway is opposed by PTEN.

The PI3K/AKT pathway is activated by the production of 3’-phosphorylated phosphoinositides (Fig. 1), and it is an important signaling pathway for MDR in a variety of cancers, such as breast cancer, leukemia, lung cancer, ovarian cancer, hepatocellular carcinoma, and melanoma^7–12^. Many types of tumors often carry at least one change in PI3K^13–17^ (Table 1). The MDR phenotype often accompanies activation of the PI3K/AKT pathway, which renders a survival signal to withstand cytotoxic anticancer drugs and enhances cancer stem cell (CSC) characteristics. However, activation of the PI3K/AKT pathway alone is not responsible for MDR in many cases, and synergistic transduction with up/downstream targets is required. Notably, the PI3K/AKT pathway is a key link that synergizes many of the targets involved in incorporating the modulation of apoptosis, cell growth, and cellular metabolism that are associated with the mechanism of MDR.

**Table 1:** Frequency of PI3K pathway alterations in cancer.

Cancer type	Altered genes		
	PIK3CA (%)	PTEN (%)	AKT1 (%)
Breast cancer	40	2–4	2–3
Endometrial cancer	68	43	3
Lung cancer	30	4	4
Squamous cell carcinoma	42.70	3	2
Ovarian cancer	68	19	27

Survival signals are different from apoptosis resistance and protect cancer cells from death. Therefore, the signaling involved in apoptosis resistance and increased survival are the main regulators of MDR in chemotherapy^18^. For example, many cell life and death signals are regulated by Bcl-2 family proteins and X-linked apoptosis proteins (XIAP), which converge in the mitochondria, where the fate of the cell is ultimately decided. Resistant cells express higher levels of Bcl-2 and/or XIAP^19^. Abnormal activation of the PI3K/AKT pathway counteracts the chemotherapeutic-induced apoptosis via the enhancing of anti-apoptotic genes, such as Bcl-2 and XIAP, and reducing of pro-apoptotic genes, such as Bax, to modulate MDR^20^.

PI3K binds to the PH domain of AKT and induces conformational changes and the subsequent phosphorylation of AKT, and activated AKT moves from the cytoplasm to the cell membrane. This cascade directly or indirectly activates its downstream molecular proteins, such as NF-κB and mTOR^21^. NF-κB is the main regulator of gene transcription and its upregulation in chemotherapy-resistant cell lines is related to treatment failure^22^. The development of MDR is related to the PI3K/AKT pathway partially due to the activation of NF-κB^23^. MDR may be due to activation of PI3K/AKT/NF-κB, which develops into the transformation of cyclin D1 and the expression of G1/S, and an acceleration of the cell cycle process^24^. The abnormal activation of PI3K/AKT/NF-κB also regulates the expression of P-gp to achieve MDR. As mentioned earlier, P13K/AKT/mTOR affected dysregulation of micro-RNA (miRNA) may also underlie MDR in tumor cells. MDR regulation of miRNA-mediated malignant tumorigenesis is an important link^25,26^. GSK-3β is a kinase that regulates proliferation in response to various stimuli. It is present in a variety of cancer types, and it involves multiple molecular pathways that evade chemotherapy, radiation therapy, and targeted therapy^27^. PI3K/AKT phosphorylates GSK-3β into an inactive form, which may cause an increase in β-catenin that translocates to the nucleus where it acts as a transcription factor to increase the expression of target genes involved in MDR^28^.

Previous studies showed that the PI3K/AKT pathway enhanced the biological basis of cancer by effectively expressing ABC transporters containing p-glycoprotein (P-gp, ABCB1), multidrug resistance-associated protein 1 (MRP1, ABCC1), and breast cancer resistance protein (BCRP, ABCG2), whose activation may reduce the response to chemotherapy drugs and enhance the drug efflux^29,30^. Due to the acidified micro-environment in tumor cells, aerobic glycolysis may confer MDR to cancer cells, which may reduce drug absorption and efficiency, and/or increase the content of intermediate metabolites^31,32^. Reducing the cellular energy of glycolytic metabolism may reduce drug resistance by reducing the outflow of drugs from cells. Notably, the PI3K/AKT pathway is involved in the regulation of aerobic glycolysis to increase energy supply, which enhances the ability of ABC transporters to excrete drugs^33^. The MDR phenotype is generally accompanied by an abnormal activation of the PI3K/AKT signaling pathway. In brief, the PI3K/AKT pathway is a key link in MDR via cooperation with upstream and downstream targets, which provides a survival signal for anti-cancer drugs.

The PI3K/AKT pathway is very complex and may have many variations and be affected by a large number of diverse inputs, which influence the MDR processes. A better understanding of PI3K/AKT synergism with other targets is important to further strengthen the rationale for cotargeted treatment strategies and combination therapy to improve the efficacy of PI3K/AKT inhibitors in the clinic.

## PI3K/AKT modulates the expression patterns of the apoptosis-related MDR process

The PI3K/AKT pathway is important for MDR in certain types of cancer, and it is a hub that influences MDR via the inhibition of apoptosis^34^. The PI3K/AKT pathway participates in the apoptotic process, which is inseparable from apoptosis-related factors, such as the Bcl-2 family and XIAP. The present study discussed how the PI3K/AKT pathway affects the MDR process in regulating apoptosis-related factors in detail.

### Bcl-2 family

Numerous studies suggested that abnormal activation of the PI3K/AKT signaling pathway contributed to the upregulation of Bcl-2 expression, which leads to apoptosis-mediated MDR in many cancer therapies^35^. The Bcl-2 protein family is a key factor in apoptosis, and it is an ancient cell suicide program that is important for cancer development and drug response^36^. Bcl-2 proteins are located on the mitochondrial membrane, including Bad, Bax, Bcl-2, Bid, and Bcl-xL, and may alter mitochondrial membrane permeability and trigger the release of cytochrome c or caspase leading to apoptotic cell death via activation of postmitochondrial caspase cascades. Anti-apoptotic proteins, such as Bcl-xL and Bcl-2, stimulate cell survival by preventing the release of cytochrome c from mitochondria, and pro-apoptotic proteins, such as Bax and Bad, induce intracellular mitochondria to release cytochrome c^37^. The balance between proteins of the Bcl-2 family plays a vital role in the apoptotic pathways induced by several foreign stimuli of PI3K/AKT^38^. The balance between these opposing apoptosis proteins, such as a simple “varistor”, regulates the sensitivity of cells to apoptotic stress^39^. The apoptotic regulator Bcl-2 is a key component of the anti-apoptotic mechanism. Bcl-2 is an oncogene that blocks mitochondrial outer membrane permeabilization and inhibits apoptosis, and it is always overexpressed in MDR of cancers. Overexpression of Bcl-2 typically results in cancer cell resistance to cancer factors, which is associated with abnormal changes in PI3K/AKT pathways^40,41^.

Activated AKT directly phosphorylates Ser136 of Bad (the target of AKT)^42^. AKT activates PAK1, which in turn phosphorylates Bad at Ser-112 and causes its release from the Bcl-xL complex to inhibit apoptosis. Activated AKT promotes cell survival via activation of Bcl-2 and inhibition of Bax^43^. The mitochondrial translocation of Bad and the physiological interaction between Bad and Bcl-xL also induce mitochondrial dysfunction, and the PI3K/AKT-mediated interaction between Bad and Bcl-xL maintains mitochondrial integrity and prevents cytochrome c efflux^44^. Therefore, PI3K activity is important for the retention of Bax in the cytoplasm, and AKT inhibits Bax translocation to the mitochondria, which is related to apoptosis-mediated MDR^45^. In summary, activation of the PI3K/AKT pathway promotes MDR via the regulation of a variety of cellular apoptotic processes (Bax/Bcl-2).

### XIAP

XIAP is an intracellular anti-apoptotic protein that plays a vital role in cell survival. It is overexpressed in many cancers, and it is involved in tumor drug resistance^46^. Notably, the PI3K/AKT pathway triggers XIAP-induced apoptosis, which may be involved in tumor cell proliferation and metastasis-mediated MDR^47^.

XIAP is one of the main inhibitors of apoptosis, and it blocks the intrinsic and extrinsic apoptosis pathways. High expression of XIAP was noted in ovarian cancer cells, and it is involved in chemoresistance^48^. Abnormal activation of the PI3K/AKT signaling pathway induces XIAP expression^49^. Kahana et al. found that downregulation of AKT reduced the expression level of XIAP^50^, which directly inhibited the activity of caspase and regulated apoptosis via a variety of pathways. XIAP is the only member of IAP famliy that blocks active caspase and inhibits apoptosis via binding to the initiator caspase (caspase-9) and the effector caspase (caspase-3)^51^. XIAP contains three baculovirus IAP repeat (BIR) domains that interact with caspase. XIAP upstream caspase-9 via its BIR3 domain, and it inhibits downstream caspase-3 and caspase-7 via its BIR2 domain^52^. XIAP is further inhibited with AKT downregulated, which eventually leads to termination of caspase activation^53^, and reversed MDR in many type of cancers. The ubiquitin E3 ligase activity of XIAP mediates the proteasome-dependent degradation of caspase, which is required for anti-apoptotic function^54^.

Previous studies demonstrated that XIAP was downstream of AKT and prevented apoptosis via the upregulation of the PI3K/AKT cell survival signaling pathway. The overexpression of XIAP using adenovirus increased phosphorylated XIAP, and increased the content of phosphorylated AKT (indicating AKT activation), which was associated with decreased MDR of cisplatin-induced apoptosis^55^. Dan et al. showed that AKT stabilized the phosphorylation of XIAP at Ser87 and protected XIAP from cisplatin-induced degradation^56^. XIAP also controlled serum starvation-induced autophagy downstream of the PI3K/AKT pathway. XIAP inhibited autophagy via regulating the level of cytosolic p53^57^. When serum induced autophagy, AKT deactivation led to dephosphorylation of XIAP, which caused XIAP to dissociate from murine double minute2 (MDM2). The kinase enhanced the ubiquitination degradation of p53 by Mdm2 and promoted the occurrence of autophagy. Therefore, the interaction of PI3K/AKT signaling pathway activation and its phosphorylation of downstream target protein of XIAP may be responsible for the mechanism of MDR due to autophagy-induced apoptosis.

## Aberrant activation of the PI3K/AKT/NF-κB and PI3K/AKT/mTOR pathways are associated with MDR

Numerous studies suggested that the PI3K/AKT pathway was the network with the highest mutation frequency in human cancers^34^. PI3K/AKT/NF-κB and PI3K/AKT/mTOR are the two main mutated pathways involved in apoptosis and tumorigenesis, and these pathways are related to MDR. The dysregulation of the major components of these two signaling pathways that leads to the activation of certain targets to promote MDR in many types of cancers is discussed below^58^.

### PI3K/AKT/NF-κB

The constitutive PI3K/AKT/NF-κB pathway is upregulated in chemoresistance-resistant cell lines and leads to treatment failure, which may be related to the inhibition of apoptosis and promotion of tumor growth by NF-κB^59^. NF-κB is a transcription factor with multiple regulatory functions, and it typically consists of a p50–p65 heterodimer^60^. In the resting state of most cells, NF-κB binds to the cytoplasmic inhibitor IκB-α. According to the classical pathway, phosphorylated IκBα rapidly degrades and releases NF-κB which is phosphorylated and translocated to the nucleus. This data suggest that the PI3K/AKT signaling pathway is involved in the modulation of IκBα via the induction of IKK phosphorylation. PI3K/AKT triggered the activation of NF-κB via enhancing the transcriptional activity of the p65 subunit^61^. Activation of PI3K/AKT inhibits apoptosis by stimulating the transactivation potential of the RelA/p65 protein. Studies also showed that the PI3K/AKT/NF-κB pathway led to activation of the AKT-mediated transcription factor c-AMP response element-binding protein (CREB) or IKK, which produced further activation of the NF-κB-exerted anti-apoptotic effects^62^. Although many studies showed that the PI3K/AKT pathway activated the NF-κB system, the main details of its molecular mechanism must be further studied^63^.

Evidence showed that the development of MDR was related to the PI3K/AKT pathway, at least in part due to the activation of NF-κB^64–66^. The MDR phenotype of breast cancer cells was associated with abnormal activation of the PI3K/AKT/NF-κB signaling pathway^67^. Constitutive and inducible activation of PI3K/AKT/NF-κB in cancer cells (such as pancreatic cancer) are closely related to tumorigenesis and promoted MDR-like cell proliferation and apoptosis^68^. Ni et al. reported that MDR of nasopharyngeal carcinoma (NPC) was reversed by inhibition of the PI3K/AKT/NF-κB signaling pathway. It primarily promoted the expression of p53 and Bax protein in NPC HK-1 cells via regulation of the PI3K/AKT/NF-κB pathway and inhibited the expression of cyclin D protein^69^. NF-κB promotes cells to pass the G1/S checkpoint by stimulating transcription of cyclin D1, which accelerates cell progression, promotes tumor growth, and leads to drug resistance^24^. The PI3K/AKT/NF-κB pathway affected the expression of Bcl-2 and caspase-3, which was cleaved by apoptotic proteins. These alterations regulated NSCLC cell growth and apoptosis to promote MDR^70^. Abnormal activation of the PI3K/AKT/NF-κB signaling pathway also regulated the expression of P-gp to achieve MDR, which is discussed in the following sections^23^. In summary, abnormality of the PI3K/AKT/NF-κB signaling pathway is a common occurrence in cancer, and it is involved in the development of MDR. It is also used as a target for reversing resistance.

### PI3K/AKT/mTOR

The PI3K/AKT/mTOR signaling pathway plays a vital role in a variety of biological and physiological processes, and survival, growth transcription, and translation, are mostly related to the occurrence of MDR^71,72^. Abnormal activation of the PI3K/AKT/mTOR pathway helps tumors to produce MDR, such as acute myeloid leukemia (AML) and ovarian cancer^73,74^.

Activated AKT directly activates mTORC1 via the phosphorylation of mTOR at Ser2448. The phosphorylation of tuberous sclerosis complex 2 (TSC2) by AKT reduces TSC1 and TSC2, which activates mTORC1. When TSC2 was inactivated by AKT, Rheb-GTP stimulated mTORC1 activity and subsequently phosphorylated 70S6K1, S6. and eukaryotic translation initiation factor 4E-binding protein 1 (4EBP1)^73,75^. Phosphorylation of 4EBP-1 also promotes the translation of mRNA encoding hypoxia-inducible factor 1α, cyclin D1, and c-Myc, which leads to angiogenesis or cell cycle progression. Abnormalities in the PI3K/AKT/mTOR pathway promote MDR via the above-mentioned mechanisms^76^. However, some studies revealed a PI3K-dependent mechanism for mTORC2 activation, which allowed mTORC2 to activate AKT in a manner that was regulated temporally and spatially by PtdIns(3,4,5) P3. AKT activates mTORC2 via the phosphorylation of mSin1 at T86, in turn the activated mTORC2 stimulates Akt through phosphorylation of Akt at S473, which forms a positive feedback regulatory loop^77,78^. Activation of mTORC2 controls cell growth via the modulation of various physiological processes, such as fat production, apoptosis, and glucose metabolism^75^. The targeting of mTORC1 is a good method to reverse MDR. However, the inhibition of mTORC1 and mTORC2 must be targeted to prevent mTORC2 from compensatory activation of AKT^79^.

The P13K/AKT/mTOR pathway is affected by miRNAs that act as negative transcriptional regulators of target genes with different potential roles in tumorigenesis and behavior^25,26^. These generally cause mutations in PIK3CA, PTEN (phosphatase and tensin homolog deleted on chromosome 10), and AKT, which are important components of the P13K/AKT/mTOR signaling pathway, and eventually lead to MDR^80^. Yue et al. found that overexpression of miR-182 reduced the resistance of trastuzumab in trastuzumab-resistant cells, partially due to inactivation of the PI3K/AKT/mTOR signaling pathway^81^. PI3K/AKT/mTOR is an essential path to MDR via the modulation of miRNAs-mediation of the tumorigenesis process of malignant cells. Similarly, long non-coding RNAs also play an important role in drug resistance. The development of MDR is promoted by high expression of XLOC_006753, and its development is activated by the PI3K/AKT/mTOR signaling pathway in GC cells^82^. LncRNA-HOTAIR silencing significantly reduced the phosphorylation of PI3K, which suggests that knocking down lncRNA-HOTAIR effectively reduced the resistance of breast cancer cells to DOX via inhibition of the PI3K/AKT/mTOR signaling pathway^83^. The PI3K/AKT/mTOR pathway is complicated. Although, it can change a lot, and it can be affected by a large number of different factors that promote MDR.

## PI3K/AKT phosphorylates GSK-3β to promote tumor proliferation related to MDR

GSK-3β is a kinase that modulates proliferation in response to various stimuli, and it is one of the subtypes of GSK-3 (a serine/threonine kinase)^84^. Dysfunction of GSK-3β is an active element in cell proliferation that is associated with cancer, and it is overexpressed in certain tumor types, including colon cancer, liver cancer, ovarian cancer, and pancreatic cancer^85,86^. GSK-3β is involved in a variety of molecular pathways, especially the PI3K/AKT pathway, to evade chemotherapy leading to MDR.

GSK-3β is generally considered a potential downstream gene product of AKT, which is a significant component of the PI3K/AKT pathway. Notably, AKT phosphorylates GSK-3β to an inactive form^28^. Phosphorylation of S9 and other residues mediates the activity of GSK-3β, which is inactivated by proteosome degradation. The consequence is that AKT is increased, which causes AKT to phosphorylate GSK-3β at S9 and its subsequent inactivation^86,87^. Activation of AKT results in decreased E-cadherin expression^88^. Because β-catenin also interacts with E-cadherin in the cell membrane, the loss of E-cadherin expression leads to an increase in cytoplasmic β-catenin, which may be rapidly eliminated by the GSK-3β-mediated process^89^. In contrast, if GSK-3β is phosphorylated or inactivated, β-catenin accumulates in the cytoplasm and transfers to the nucleus, where it interacts with cytokines, such as lymphokine active factors^90^. Acceleration of the transcription process and upregulation of c-Mye, c-Jun, and cyclinD1 expressions may promote tumor proliferation and increase the expression of target genes, such as MDR1 and survivin, which further leads to MDR^28^.

Gao et al. found that GSK-3β regulated cell viability and induced MDR via the PTEN/PI3K/AKT signaling pathway. The results indicated that GSK-3β induced PTEN phosphorylation and led to AKT activation in cancer cells. GSK-3β was a negative regulator of the PI3K/AKT signaling pathway, and GSK3β-mediate phosphorylation or PTEN inactivation resulted in depression of the PI3K/AKT signaling by macrophages/microglia^91^. AKT inhibited the expression of GSK-3β, which increased the expression of PTEN, and played a role in the conversion of PIP3 to PIP2, which regulated the activity of AKT via a positive feedback loop. However, the conservative mechanism of MDR in breast cancer has not been confirmed^92^.

In conclusion, GSK-3β exerts the dual role of drug resistance as a promoter or tumor suppressor, interacts with the PI3K/AKT pathway, regulates tumor proliferation and apoptosis, and ultimately relates to MDR.

## PI3K/AKT participates in MDR processes via induction of the ABC transporter

**Fig. 2:**
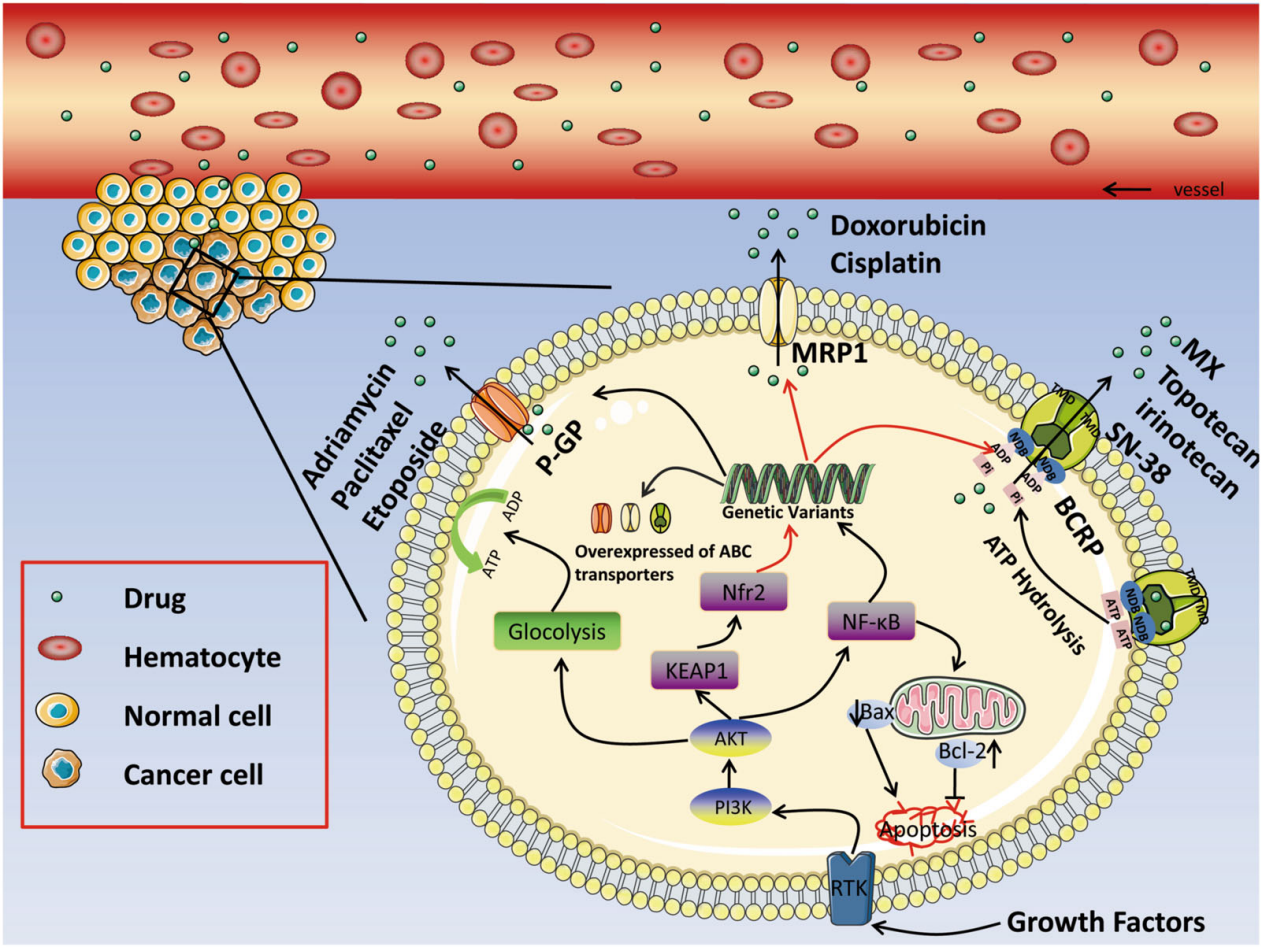
The PI3K/AKT pathway regulates the gene expression of ABC transporters by activating downstream targets, such as NF-κB and Nfr2, resulting in over-expression of P-gp, MRP1, BCRP. Thus, accelerating the transport of anticancer drugs by ABC transporter to the outside that leading to MDR.

Cancer cells exhibit MDR to anticancer drugs via several mechanisms. Obviously, one of the most major mechanisms of MDR is the overexpression of ABC superfamily transporters^93^. Forty-eight human ABC genes were discovered and divided into seven different subfamilies (ABCA–ABCG)^94^. P-gp, MRP1, and BCRP, are the three major ABC transporters that confer many of the structural diversities related to anticancer drug resistance^95^. A number of chemotherapeutic agents, such as paclitaxel, cisplatin, topotecan, irinotecan, and SN-38 are transported by these transporters. Recent studies indicated that the PI3K/AKT signaling pathway was involved in the formation of MDR by inducing the expression of membrane transporters^96^. Once activated, the PI3K/AKT pathway becomes the basis of cancer biology and enhances drug efflux by efficiently expressing ABC transporters and reducing the response of chemotherapeutic drugs, which further caused MDR^29,30^ (Fig. 2).

### P-gp

P-gp is one of the first members of the ABC transporter. It was isolated by Juliano and Ling from colchicine-resistant Chinese hamster ovary cells^97^. Activation of the PI3K/AKT signaling pathway modulated the expression of P-gp, which extruded toxins and xenobiotics from cells and led to MDR^23^.

P-gp as a unique transporter that is expressed by two different linked genes in mouse, such as MDR1a and MDR1b, but the human MDR1 gene is responsible for P-gp production^98^. The PI3K/AKT kinase pathway may participate in the regulation of P-gp-mediated MDR, which is a major obstacle to the successful treatment of acute megakaryoblastic leukemia (AML)^99^. Previous reports showed that P-gp resisted apoptosis by transporting key caspases out of cells or inhibiting the activity of caspase. During apoptosis, cleavage occurs in a caspase-dependent manner, which indicates the complexity of the apoptosis and MDR signaling network^100^.

Abnormal activation of the PI3K/AKT signaling pathway mediated the expression of P-gp, which is connected to the control of the MDR phenotype^23^. Ganesan Muthusamy et al. found that ferulic acid contributed to the reversal of the MDR via suppression of P-gp expression and the inhibition of PI3K/AKT/NF-κB^101^. Xie et al. used LY294002 to inhibit the PI3K/AKT signaling pathway and showed that PI3K/AKT regulated the transcription of MDR1 in gastric cancer cells at the mRNA and protein levels^102^. Other studies showed that activation of the NF-κB system by the PI3K/AKT signaling pathway resulted in the increased transcription of target genes, such as ABCB1^103^. The AKT/NF-κB signaling pathway is the main mechanism by which CD133 regulates P-gp expression in colorectal carcinoma^104^. The NF-κB pathway drives MDR1 expression using the common agonist phorbol ester 12-O-tetradecanoyl-13-acetate (TPA). LY294002 treatment down regulated the expression of NF-κB, which indicates that the inhibition of the PI3K/AKT signaling pathway modulated the downregulation of MDR1 expression^105,106^. The modulation of P-gp phosphorylation by AKT kinase or the direct interaction of P-gp with inhibitors cannot be excluded^107^. In conclusion, abnormal activation of the PI3K/AKT signaling pathway mediated the expression of P-gp and led to MDR.

### MRP1

MRP1 is one of the earliest described ABC transporters, and it is expressed in the plasma membrane of normal cells and cancer cells. MRP excretes physiological toxins and various agents from cells, which reduces cytotoxicity^108^. Study showed that inhibition of the PI3K/AKT signaling pathway led to a down regulation of MRP1 and a reversal of MDR in cancer cells^109^.

MRP1 is primarily expressed in physiological disorders, such as the blood–brain barrier and may limit drug uptake and retention^110^. Activation of the PI3K/AKT pathway promotes via MDR by regulation of the expression of a variety of cellular processes, including apoptosis (Bax/Bcl-2) and drug transport (MRP1)^111^, where nuclear factor erythroid-derived 2 (Nrf2) is a nuclear regulator of MRP1, and it is part of the reason for MDR. Yao et al. found that MDR was reversed by interfering with the PI3K/AKT/Nrf2 pathway, which led to MRP1-mediated drug outflow^112^. MRP1 inhibition depended on the inhibition of AKT phosphorylation and nuclear Nrf2. Gao et al. showed that vielanin P (VP) reduced PI3K110α. However, in the presence of siNrf2, the levels of Nrf2 and MRP1 were not further reduced. These results indicated that VP regulated MRP1 via PI3K/AKT/Nrf2. These convincing results suggested that the PI3K/AKT/Nrf2 pathway stimulated MRP1 and participated in the reversal of VP-mediated MDR cancer^113^. The PI3K/AKT signaling pathway inhibitor LY294002 blocked the drug transport of HT29RDB overexpressing MRP1 protein in colon cancer cells^114^. Therefore, the transport activities of P-gp and MRP1 may have a common regulatory mechanism involved in the PI3K/AKT pathway, and their inhibitors similarly affected resistance based on P-gp and MRP1^115^. In summary, aberrant activation of the PI3K/AKT signaling pathway is vital for MRP1. Inhibition of the PI3K/AKT pathway is a valid method for mediating MRP1 to reverse MDR.

### BCRP

BCRP is encoded by ABCG2, which is one of the major transporters involved in xenobiotic efflux^116^. An increasing number of renal chemotherapeutic drugs (tyrosine kinase inhibitors) are described as BCRP substrates and can regulate their activity via the PI3K/AKT signaling pathway^117,118^. AKT mediates the regulation of ABCG2 function and its localization on the plasma membrane. This regulation is not a limiting factor in cancer development, but a potential response of the tumor to cancer therapy. After activated, this pathway was less sensitive to chemotherapeutic drugs, which contributed to MDR^119^.

Other members of the BCRP and ABCG families are substantially different from the prominent ABC transporters and are referred to as “semitransporters”^120^. Intracellular receptors that sense exogenous hormones and stimuli include estrogen (ER) and aryl hydrocarbons (Ahr). These stimuli trigger transcriptional responses through AKT, Kelch-like ECH-related protein 1 (KEAP1) and Nrf2, which upregulate the expression of BCRP. Upstream or downstream inhibition of epidermal growth factor receptor (EGFR/HERB)-dependent signaling regulates the AKT cascade and causes BCRP inhibition or activation. The PI3K/AKT signaling pathway regulated BCRP expression, and BCRP may protect multiple myeloma (MM) cells from the cytotoxicity of chemotherapeutic drugs by phosphatase and tensin homolog expression via a potential negative feedback loop of BCRP, and it is modulated by PTEN-mediated PI3K/AKT pathway regulation in NCI-H929 cells^121^. In addition, CSCs often exhibits a high ABCG2 transporter activity, the ratio of lateral cell (SP) cells in MM patients, ABCG2 expression and activation of the PI3K/AKT pathway are all positively correlated with disease progression. These studies demonstrate the key role of BCRP and PI3K/AKT pathway in controlling cancer stems and provides new strategies for the targeting of BCRP and the PI3K/AKT pathway to treat MDR.

## The PI3K/AKT pathway is involved in the regulation of aerobic glycolysis to increase the energy supply leading to MDR

Metabolic reprogramming is the most important difference between cancer cells and healthy cells, which is the major metabolic phenotype of cancer^122^. For example, cancer cells may show additional metabolic reprogramming during chemotherapy to gain resistance to antineoplastic drugs^123^. The best feature of these metabolic changes is the Warburg effect, in which cancer cells tend to rely on glycolysis for energy and anabolic metabolism, even if oxygen levels are sufficient for oxidative phosphorylation. The PI3K/AKT pathway is a regulator of aerobic glycolysis and central glucose metabolism^124^. It confer advantages on MDR due to the acidification of the intracellular microenvironment, which may reduce drug absorption and efficiency, and/or an increase in intermediate metabolites^125,126^. Because cell growth depends on the biosynthesis of cellular building blocks produced by metabolic intermediates, it is easy to understand the seemingly contradictory use of this fuel^127^.

The PI3K/AKT signaling pathway regulates glycolysis via the upregulation of enzymes that induce stimulation of glycolytic enzymes, such as glucose transporters (GLUTs) and phosphofructokinase (PFK)^128,129^. GLUT1 is the main glucose transporter, and it is overexpressed in many cancer types. The upregulation of PI3K/AKT enhances its expression^130^. PI3K-dependent AKT direct phosphorylation and activation of PFK2 improves the production of fructose-2,6-diphosphate and ultimately activates the glycolytic rate-restricting enzyme PFK1^131^. The underlying mechanisms of these effects may be related to the PI3K/AKT pathway.

The PI3K/AKT pathway modulates the regulation of aerobic glycolysis in cancer cells^132^. The most upregulated pathway in K562/ADM cells was the PI3K/AKT signaling pathway, which was related to the reprogramming of glucose metabolism and the occurrence of MDR. The PI3K/AKT pathway was upregulated in cancer cells, which may lead to aerobic glycolysis and MDR enhancement^31^. The increased expression of GLUT4 confirmed the excessive activation of the PI3K/AKT/mTOR signaling pathway in cancer cells, which may enhance the ability of MDR cells to rapidly transport and consume glucose via glycolysis to produce ATP. The inhibition of glycolysis led to a depletion of ATP and led to the blockade of ATP-dependent drug outflow function that caused P-gp, which may be a strategy to reverse MDR^6^. Therefore, the PI3K/AKT pathway can regulate aerobic glycolysis, and it is the key energy supplier to the metabolic adaptation of cancer cells for MDR.

## Prospective and conclusion

MDR is a serious obstacle to chemotherapy because it enables tumor cells to escape chemotherapy even at high doses, and it promotes tumor metastasis and recurrence. MDR is caused by multiple mechanisms. Impediments to apoptosis, reducing drug accumulation, alterations in cell cycle and enhancement of the tumor energy supply are the main reasons for MDR. Increases in DNA repair capacity, checkpoint changes, the presence of CSCs in tumors, gene mutations (PTEN mutations or disappearance, mutations in the TP53 gene), changes in drug target separation and epithelial–mesenchymal transition are also related to MDR^133–135^. These mechanisms must be carefully discussed in the future.

The present review showed that the PI3K/AKT pathway is a key link in the regulation of cancer MDR, and inhibition of this pathway may be an important way to solve tumor resistance. Currently PI3K/AKT inhibitors have been successfully used to enhance the sensitivity of cancer cells to drug-induced apoptosis^136^. For example, econazole was a new type of PI3K inhibitor, which inhibited the PI3K/AKT signaling pathway and reversed the resistance of breast cancer cells to doxorubicin^137^. Some studies showed that phenybutyl isoselenocyanate (ISC-4) treatment significantly inhibited PI3K/AKT activation in a dose-dependent manner, and ISC-4-mediated p-Akt inhibition resulted in primary AML (CD34+) stem cell apoptosis and enhanced the efficacy of cytidine^138^. Therefore, PI3K/AKT inhibitors have a central role in the development of novel strategies to overcome MDR. However, the effect of simple inhibitors in the clinical application of drugs is not ideal. Many clinical trials using PI3K, AKT, mTOR, or dual inhibitors in combination with endocrine or chemotherapy are underway. The usage of PI3K/AKT inhibitors in combination with other anti-cancer drugs requires in-depth research to provide a more effective means for clinical tumor treatment.

**Fig. 3:**
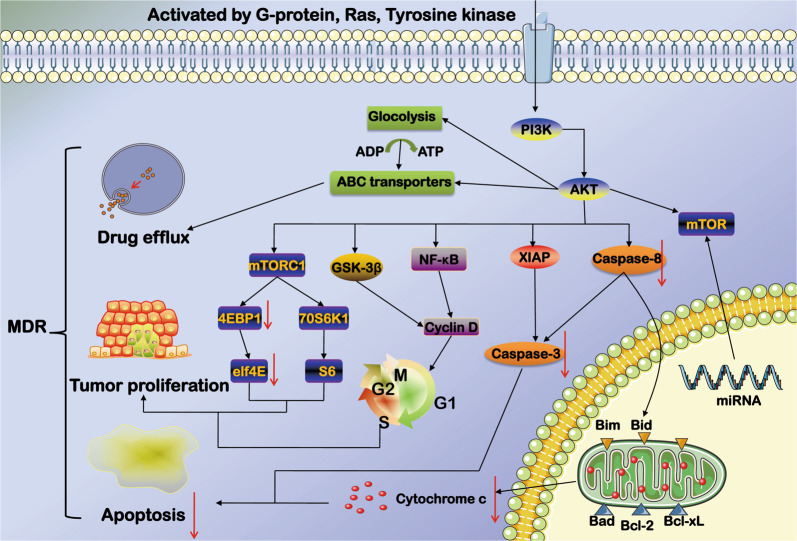
A dysfunctional PI3K/AKT pathway serves as a hub to regulate many cellular processes that are involved in MDR including apoptosis, ABC transporter activity, mTOR pathway, and tumor metabolism. *Note*: The PI3K/AKT pathway enhances drug efflux through effective expression of ABC transporter. Aerobic glycolysis is the key energy supplier to the metabolic adaptation of cancer cells for MDR. The PI3K/AKT pathway affects tumor proliferation by regulating mTOR, GSK-3β, and NF-κB. The PI3K/AKT pathway triggers XIAP to suppress the activity of caspase-3 inhibiting apoptosis. The decrease of caspase-8 will reduce migration of Bid to mitochondria, and slim down the activation of Bax and Bim, which lead to the decrease of mitochondrial membrane permeability (MOMP) and the release of cytochrome c. Caspase-8 also regulates the expression of caspase-3 to achieve cell apoptosis. The P13K/AKT/mTOR may also underlie MDR in tumor cells through inducing dysregulation of miRNA.

The present review systematically summarized that a dysfunctional PI3K/AKT pathway serves as a hub to regulate many cellular processes that are involved in MDR^3^ (Fig. 3). It can avoid MDR by inhibiting the PI3K/AKT pathway and related target proteins. It is possible to create an armory that overcomes MDR in different clinical situations. For example, sustained PI3K/AKT/mTORC1 activity may also be attributed to changes in miRNA expression and can induce many epigenetic changes that perpetuate MDR, which was discussed in this review. However, no therapeutics targeting specific miRNAs have made it into the clinic. miRNA inhibitors can also be expected to treat MDR by indirectly reducing the activity of the PI3K/AKT pathway. However, due to the complexity of these elements, direct targeting must be carefully studied.
